# Towards A Framework for Implementing Remote Patient Monitoring From an Integrated Care Perspective: A Scoping Review

**DOI:** 10.34172/ijhpm.2023.7299

**Published:** 2023-03-01

**Authors:** Rafael Miranda, Mónica Duarte Oliveira, Paulo Nicola, Filipa Matos Baptista, Isabel Albuquerque

**Affiliations:** ^1^Centro de Estudos de Gestão do Instituto Superior Técnico (CEG-IST), Instituto Superior Técnico, Universidade de Lisboa, Lisboa, Portugal; ^2^Enterprise Services, Siemens Healthineers Portugal, Lisboa, Portuga; ^3^iBB - Institute for Bioengineering and Biosciences and i4HB - Associate Laboratory Institute for Health and Bioeconomy, Instituto Superior Técnico, Universidade de Lisboa, Lisboa, Portugal; ^4^Instituto de Medicina Preventiva e Saúde Pública, Faculdade de Medicina, Universidade de Lisboa, Lisboa, Portuga; ^5^Comprehensive Health Research Center (CHRC), Universidade NOVA de Lisboa, Lisboa, Portugal

**Keywords:** Remote Patient Monitoring, Telehealth, Integrated Care, Scoping Review, Healthcare Organization

## Abstract

**Background:** Remote patient monitoring (RPM) has been increasingly adopted over the last decade, with the COVID-19 pandemic fostering its rapid development. As RPM implementation is recognised as complex and highly demanding in terms of resources and processes, there are multiple challenges in providing RPM in an integrated logic.

**Methods:** To examine the structural elements that are relevant for implementing RPM integrated care, a scoping review was conducted in PubMed, Scopus, and Web of Science, leveraging a search strategy that combines terms relative to (1) conceptual models and reallife initiatives; (2) RPM; and (3) care integration.

**Results:** 28 articles were included, covering nine conceptual models and 19 real-life initiatives. Eighteen structural elements of RPM integrated care implementation were identified among conceptual models, defining a structure for assessing real-life initiatives. 78.9% of those initiatives referred to at least ten structural elements, with *patient education and self-monitoring promotion, multidisciplinary core workforce, ICTs (information and communications technologies) and telemonitoring devices,* and *health indicators measurement* being present in all studies, and therefore being core elements to the design of RPM initiatives.

**Conclusion:** RPM goes far beyond technology, with underlying processes and involved actors playing a central role in care provision. The structural elements identified can guide RPM implementation and promote maturity in adoption. Future research may focus on assessing design completeness, evaluating impacts, and analysing related financial arrangements.

## Introduction

 In a 2014 essay on the main trends in healthcare provision for 2020 and beyond,^1^ the Deloitte Centre for Health Solutions envisaged a healthcare system that integrates home care and technology. Hence, “the home is where much of the medical care takes place. (…) The ubiquity of digital communication means that many doctor-patient contacts are now virtual and deliver care to the patient in their home. Specialist hospital treatment is reserved for trauma and emergency surgery; (…) while chronic and long-term conditions are managed in the community.” While this essay was meant to be bold and provocative, this prediction captures the paradigm that healthcare has had to adapt to as a result of the COVID-19 pandemic.^2-4^

 Out of necessity, remote care initiatives, mainly conceived as small pilots,^5,6^ overcame existing barriers and asserted themselves as viable solutions to deliver care that would otherwise remain unprovided.^7-10^ Remote patient monitoring (RPM) can be defined as “a mode of healthcare delivery that gathers and integrates patient data outside of traditional healthcare settings, allowing providers to track, assess, and engage patients regardless of location.”^11^ RPM can thus constitute an alternative (but also a complement) to conventional care, with potential social and economic value for both patients and providers. The latter can follow multiple patients simultaneously, monitor their vital signs and reported symptoms, provide educational materials that promote health literacy and self-care, and adapt care delivery to meet patients’ needs better. In return, patients can receive care in a more comfortable and familiar environment, avoiding exposure to increased and unnecessary risks (eg, hospital-acquired infections) and psychological distress.^12-15^

 While RPM was far from widespread before the COVID-19 pandemic,^16,17^ afterward, its implementation rapidly responded to emerging adversities,^18^ but faced design limitations.^19-21^ To our knowledge, there is still a considerable gap in implementing RPM within a continuum of care, which requires coordination and communication between actors and full consideration of involved technology, procedures, and outcome measurement.^22-24^

 To fill this gap, this scoping review^25^ aims to examine what structural elements need to be considered to promote an integrated care implementation of RPM initiatives. Existing literature on conceptual models and real-life initiatives will be assessed, allowing a comprehensive appraisal of the state-of-the-art. To ensure the assessment of the integrated care perspective and enable the identification of the main structural elements encompassed, the **S**ustainable int**E**grated chronic care mode**L**s for multi-morbidity: delivery, **FI**nancing, and performanc**E** (SELFIE) framework for integrated care for multi-morbidity^26^ is adapted and applied to the context of remote care provision.

## Methods

###  Search Strategy and Study Selection

 A search for scientific literature was conducted on June 8, 2021, in the following bibliographic databases: PubMed, Scopus, and Web of Science. Articles were searched according to a comprehensive search protocol based on keyword combination and considering terminology variations and alternative spellings. This protocol was applied to the title and abstract fields of articles published since 2010, ensuring a thorough analysis of the state-of-the-art over the last decade and over the pandemic period (period of high proliferation of telehealth^27^). The search algorithm combined terms referring to (*a*) conceptual models, programs, and initiatives; (*b*) RPM and telemonitoring (TM); and (*c*) care integration and continuity of care. The search protocol for article identification can be consulted in Table S1 of Supplementary file 1.

 The existence of duplicates was first verified using the Mendeley’s “Check for Duplicates” tool, with duplicate references being merged. The same verification process was then performed manually. Hereafter, article screening was conducted in two steps – the first on the title and abstract fields, to identify articles relevant for retrieval, and the second on full-text eligibility for review inclusion.

 During the first step, conducted by RM, all references whose title and abstract suggested configuring (even if tenuously) an RPM implementation framework or program and following a care integration logic were sought for retrieval. The second step was predominantly conducted by RM and validated by MDO whenever the decision to include an article was not immediately clear.

 Exclusion criteria were defined according to Armstrong et al^28^ methodology for developing scoping reviews. Articles were excluded if (*a*) full-text was not written in English; (*b*) literary object was an editorial, letter to the editor, commentary or conference abstract; (*c*) report did not describe an RPM intervention^11^; or (*d*) report failed to present a framework or program of integrated implementation of RPM (ie, report must describe both human and non-human elements intervening in a coordinated RPM care delivery, with the patient playing an active role managing its health status). For instance, reports describing solely videoconferencing (VC), telephone-based monitoring, electronic medical record (EMR) integration, or telerehabilitation were not considered. Additionally, reports describing self-management or information-gathering solutions, with no patient-provider communication nor coordination between providers, were also excluded.

 To meet the main objective of this scoping review and the logical distinction between assessed bodies of literature, the selected articles were divided into two groups – *conceptual models* and *real-life initiatives* (eg, case studies, clinical trials). Conceptual models were analysed first and initiatives second, in the same order in which the results are presented.

 Preferred Reporting Items for Systematic Reviews and Meta-Analyses extension for Scoping Reviews (PRISMA-ScR)^25^ guidelines were followed in the development of the study.

###  Assessing the Integrated Care Nature of the Studies – the SELFIE Framework

 The SELFIE framework^26^ was built upon existing models for integrated and person-centred care (such as the Chronic Care Model, the Guided Care Model, and the Development Model for Integrated Care), and enriched through a highly comprehensive scoping review and consultation of experts from 8 European Union countries and representatives of relevant stakeholder groups (ie, patients, partners, professionals, payers, and policy-makers). SELFIE was set to target multi-morbidity and capture complexity in the delivery of care; it provides a structure of interconnected concepts that can be applied to guide the development, implementation, description, and evaluation of integrated care programs.^26^

 Under SELFIE, the holistic understanding of the person with multi-morbidity and respective environment is placed at the centre of the framework, interacting with surrounding elements pertaining to integrated care. These elements are further grouped according to six components – *service delivery*, *leadership *&* governance*, *workforce*, *financing*, *technologies *&* medical products*, and *information *&* research* – and, within each component, the distinction is made between the micro (comprised elements), meso (coordination) and macro (legislation and policies) levels. Transversal to all components and levels is a *Monitoring* component to stimulate continuous improvement in the remaining ones. Each component and subsequent levels are described in detail in Leijten et al.^26^

 In the context of the present study, the SELFIE framework was used as a starting point to systematically identify and describe the elements of care integration present in included studies. As we intended to identify the structural elements for integrated care implementation of RPM initiatives, the macro level of the SELFIE components was not considered, since legislative and policy issues were defined as outside the scope of analysis.

 The results from conceptual model studies’ analysis are presented along SELFIE care integration components, allowing to group identified elements of RPM care integration. However, we did not differentiate identified elements at the micro or meso levels of the framework, as we believe this adds a layer of unnecessary complexity to our work.

## Results

###  Literature Search Results

**Figure 1:**
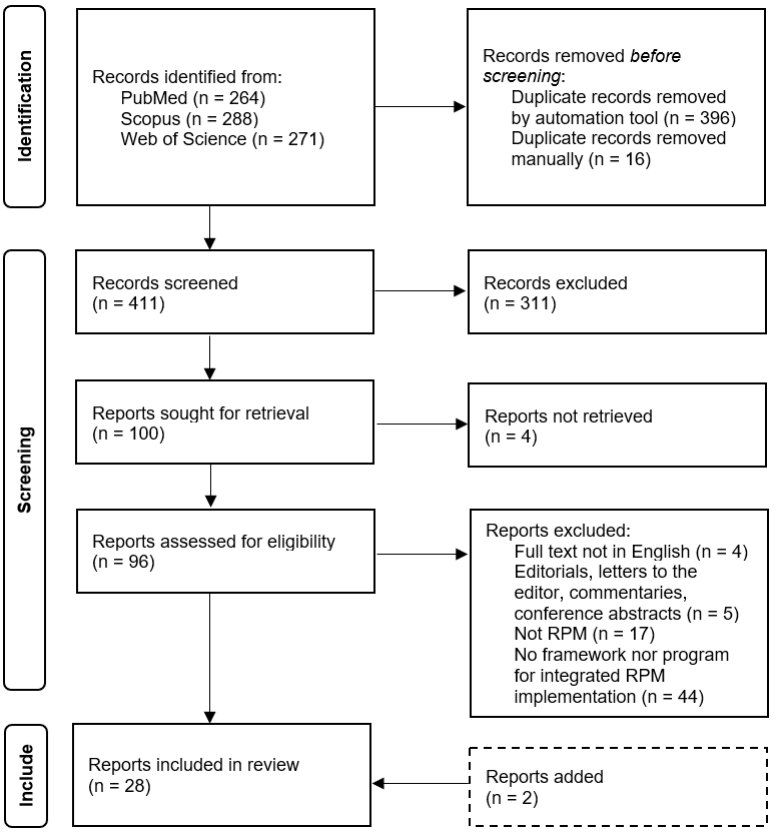
PRISMA-ScR Flow Diagram of the Study Selection and Exclusion Criteria Used in the Study. Abbreviations: PRISMA-ScR, Preferred Reporting Items for Systematic reviews and Meta-Analyses extension for Scoping Reviews; RPM, remote patient monitoring.

 A literature search conducted on the specified databases identified 823 records meeting the described keyword combinations. After duplicate removal, 411 articles remained for title and abstract screening, leading to the exclusion of 311 references. Not being possible to retrieve full text for 4 records, 96 articles were assessed for review inclusion eligibility. Exclusion criteria application led to rejecting 70 articles – 4 not written in English language, 4 conference abstracts, 1 letter to the editor, and 61 out of scope. 2 further articles were deemed relevant (identified through updated search) and added; thus, 28 studies were included in the scoping review – 9 *conceptual models* and 19 *real-life initiatives* of RPM-based integrated care implementation. The identification and screening process conducting to this final sample is depicted in Figure 1.

###  Study Characteristics

 The 28 selected studies spanned across 10 different nations (ie, country where the study was conducted or, if not stated, first author institution’s country) – USA (13 studies),^11,14,29-39^ Italy (3),^40-42^ Netherlands (3),^43-45^ Canada (2),^46,47^ France (2),^48,49^ Austria,^50^ Finland,^51^ Germany,^52^ Norway,^53^ and Scotland.^54^

 Concerning the diseases, conditions or the specific groups of patients addressed, these selection of studies focuses predominantly (71.4%) on chronic conditions (20) ie, on generalized chronic patients’ management,^43,46,49^ heart failure (HF),^29,36,45,50-52,54^ chronic obstructive pulmonary disease (COPD),^40,45,53^ cancer,^37,39,48^ diabetes,^33^ amyotrophic lateral sclerosis,^44^ dementia,^41^ infants with single ventricle physiology,^34^ and patients possessing cardiac implantable electronic devices (CIED).^42^ Other non-chronic conditions or specific groups of patients addressed are COVID-19 patients,^11,14,47^ prenatal care,^14^ older adults,^30^ patients with mental illnesses (post-traumatic stress disorder^35^ and depression^32^), lung transplant^38^ and post-acute care^31^ patients.

###  Conceptual Models for Integrated Care Implementation of Remote Patient Monitoring

 Articles included in conceptual models’ analysis can be grouped into three categories, according to the main purpose of the study: (*a*) conceptual extension of an implemented initiative^46,48,50^; (*b*) expert recommendations^30,36,39,40,49^; and (*c*) RPM-specific business model proposal.^43^

 Conceptual extensions derive from projects already implemented, pinpointing challenges and/or inefficiencies incurred in the past. Ferrua et al^48^ outlined an RPM system to improve oral medication cancer therapy. Gordon et al^46^ conducted interviews with patients and health professionals to inform the development of TM for multi-morbidity chronic disease management (a follow-up article was published recently,^55^ which we consulted for further details). Modre-Osprian et al^50^ developed a concept combining closed-loop monitoring with a collaborative network for HF management.

 Studies reporting recommendations from RPM experts rely on scientific evidence and/or lived experiences to formulate best implementation practices. Although these articles do not present a model *per se*, the detail and scope were considered sufficient to infer a framework for implementation. Aronoff-Spencer et al^39^ describe a participatory approach to engage stakeholders in designing a framework for remote care of rural patients experiencing distress during cancer treatment. Chen and Levkoff ^30^ outline recommendations on human-computer interaction within TM care for older adults. Dimengo and Stegall^36^ propose recommendations on team-based care TM in patients with HF, leveraging self-efficacy and behaviour change strategies. Donner et al^40^ present a summary of a workshop on telemedicine use to facilitate the integrated care of COPD. Bourret and Bousquet^49^ propose an integrated health system combining information and communication technologies (ICTs), shared-decision making and primary care services to treat noncommunicable diseases.

 Grustam et al^43^ compared *business-to-business* (B2B) and *business-to-consumer* (B2C) models for TM from the perspectives of both activity system theory and transaction cost theory.

 Conceptual model articles were examined considering SELFIE’s components of care integration, according to which the results of this analysis are presented below.

####  Holistic Understanding of the Individual in His/Her Environment (Individual & Environment)

 Assessing patients’ perspectives throughout program design^39,46,48,55^ allows providers to adapt the intervention to the end-users’ technological and self-management capabilities, as well as understand possible reasons for dissatisfaction with conventional care.

 Technology Acceptance Model and digital divide theory offer insights into the factors that influence the willingness-to-accept technology for health management.^30^ Individuals, particularly older adults, are often influenced by affordability, ease to assemble/operate, or user-friendliness in perceiving benefit.^36^ Physical impairments can also reduce one’s confidence to operating technology.^30,40^ Geography, community context and language are also major determinants of technological acceptance.^39,46,55^

####  Service Delivery

 Patient education and self-monitoring promotion are transversal themes among studies. During enrolment and throughout intervention phases, individuals (and caregivers) are instructed on correctly using TM equipment, educated about their condition, and presented with self-management opportunities,^50^ contributing to increase therapeutic adherence.^48^ Online classrooms, social networking, simulation/gaming,^40^ self-care knowledge quizzes, and support group sessions^36^ are relevant activities for promoting self-management and behaviour change.

 Tailoring service delivery to the patients’ complex needs (eg, considering the challenges of multi-morbidity^43,48^) and dynamic evolution of their health status also plays an important role in improving care quality and engagement.^46,49,50^ As therapy progresses, improvements in patient’s health status and self-management capacity may not justify maintaining the intervention as is. Thus, Modre-Osprian et al^50^ introduces the concept of “dynamic trajectory of illness,” according to which the individual can engage in four configurations of RPM – *collaborative TM* (leveraging multidisciplinary intensive care coordination), *“classical TM,” home-care monitoring* and *self-management*.

 Grustam et al^43^ presented two models for TM care delivery – the B2B model, describing a *hospital-to-home* (H2H) care delivery, since communication takes place between the patient’s home and a hospital-located telehealth team using ICTs, during outpatient rounds (ie, vital signs are assessed remotely against personal goals/thresholds); and the B2C model, describing a *high-touch-high-tech* (2HT) approach, based on a TM centre that coordinates stakeholder interaction, where *telenurses* act both as “healthcare navigators” and “personal health coaches,” aided by personalized, smart algorithms for patient monitoring. H2H care delivery is set for discharged patients after an urgent episode, while 2HT delivery is particularly relevant for chronic disease management.

####  Leadership & Governance

 A shared decision-making culture, involving all stakeholders (including patient and caregivers), is considered one major feature of integrated care delivery.^40,49,50^ Coordination of micro-level decision-making processes (ie, patient-provider interaction) is usually ensured by a coordination pivot (eg, case managers, nurse navigators). In most studies, this role is performed by nurses,^43,46,48,50,55^ who conduct activities from answering patients’ proactive contacts^48^ to leading the entire care model, undertaking all medical coordination decisions.^50^

 Higher coordination is achieved through a program coordinator, responsible for orchestrating all stakeholders and partners, so they can efficiently work together.^50^ Grustam et al^43^ describes two program coordination types – between places of activity (*stages*) and between participants in the activity (*actors*). Within *stages*’ coordination, care coordinators are needed in both interacting sites (eg, nurse at the hospital, caregiver at home). In *actors*’ coordination, TM nurses are the solo care delivery coordinators, managing person and institutional interdependencies between providers.

####  Workforce

 Caregivers, nurses (specialists or not) and physicians (specialists or general practitioners) are the main actors responsible for monitoring and acting upon clinical deterioration, constituting the core RPM workforce across studies. Other professionals may also play an important role in the intervention, such as other specialty physicians (eg, in the context of HF, endocrinologists, pulmonologists, or psychologists^50^), dieticians, social workers^46,55^ and pharmacists.^43,49^

####  Financing

 Modre-Osprian et al^50^ suggests that RPM configurations such as *collaborative TM* and *“classical TM”* could be covered by public funds, while *home-care monitoring* and *self-management *would be delivered by private providers, stating, however, that such public-private arrangements might complicate care continuity.

 Not only in care delivery and governance, B2B and B2C models also differ in financing and payment flows. B2B models imply commercial transactions between two businesses (eg, a TM equipment manufacturer and a hospital). In contrast, transactions are processed directly with the end-user in B2C models (eg, TM centre and patient). New reimbursement strategies may be needed to cover possible out-of-pocket expenses, such as (*a*) reimbursement by government/insurer to patients, (*b*) payment by government/insurer to TM centre for a patient cohort, or (*c*) payment by informal caregivers to TM centres.^30,43^

####  Technologies & Medical Products

 In most studies, patient portals, mobile-based apps, and TM devices are core technological components. Using a smartphone, tablet or web-based platform, one can acquire vital signs through TM devices,^43^ send messages, collect electronic patient reported outcomes (e-PROs),^39^ schedule appointments, provide educational materials, storage exam results, and/or check/deliver reminders.^48^ Aronoff-Spencer et al^39^ and Chen and Levkoff ^30^ draw recommendations on interface design: (*a*) compatibility with existing hardware; (*b*) simplified design, based on existing or low-cost-low-training devices; (*c*) critical judgement in deciding what information to include; (*d*) automated and unobtrusive data collection and transmission (eg, Bluetooth (BT)-enabled); (*e*) use of audio-visual communication.

 At a higher level of technological coordination, Modre-Osprian et al^50^ proposes a health data centre, an interoperable information system capable of processing and analysing data, detecting upcoming adverse events and producing action triggers, through artificial intelligence-driven decision support systems.

####  Information & Research

 Data visualization can be supported by clinical dashboards that provide comprehensive follow-up of patients’ health status in a user-friendly manner.^39,43,50^ Dashboards should include both manually- and automatically-gathered data, and be accessible to all relevant stakeholders involved.^40^

 Aspects to be monitored are condition-dependent, thus shall be defined according to target population’s needs. Blood pressure (BP), heart rate, electrocardiogram (ECG), weight, glycaemia, dyspnoea, blood oxygenation, sleep patterns, anxiety, or physical activity are examples mentioned across studies. Relevant information which cannot be measured through TM devices can be assessed by e-PRO questionnaires.^36,40^

####  Monitoring

 Outcome measurements^36,39^ such as number of admissions/readmissions, emergency department visits, inpatient length of stay, all-cause mortality, changes in patterns of use, health-related quality of life variation, overall patient satisfaction, user experience and system usability scores, cost saving for patients and providers, and return on investment are only some relevant performance indicators to consider in RPM.

Table 1 presents a summary of the good practices and/or recommendations inferred from studies’ analysis, grouped by 18 distinct elements of integrated care implementation of RPM, in turn grouped according to the components of integrated care proposed by the SELFIE framework.^26^

**Table 1:** Elements of RPM Integrated Care Implementation That Emerged From the Analysis of the Conceptual Model Studies

**SELFIE Framework’s Components**	**Elements of Integrated Care Implementation of RPM**	**Code**	**Good Practices and/or Recommendations From Studies**
Individual & environment	Collaborative design process	(a)	Conduct interviews with patients and caregivers to develop end-user-tailored interventions^39,46,48^
			Apply co-design techniques to engage all relevant stakeholders in the cocreation process^39^
	Patient-centred implementation	(b)	Technology Acceptance Model and digital divide theory^30^
			Consider the impact of physical impairments on technology usage^30,40^
			Consider in-home limitations and difficulties to assemble/operate TM equipment^36^
			Geographical and community context role in technological acceptance and digital literacy^39,46^
Service delivery	Patient education and self-monitoring promotion	(c)	Continuously instruct patients (and caregivers) on how to correctly use the TM equipment^30,36,39,40,43,46,48–50^
			Promote education on the patient’s health condition and on self-monitoring opportunities^48,50^
			Promote online classrooms, social networking, online simulation/gaming, quizzes to assess self-care knowledge, support group sessions^36,40^
	Multi-morbidity care	(d)	Consider adjacent health manifestations when designing care delivery pathways^49,50^
			Micro-level coordination role in improving patient navigation within this complex care setting^43,48^
	Dynamic trajectory of illness	(e)	Individuals can engage in different RPM levels according to their needs^50^
	*H2H *vs *2HT*	(f)	Communication takes place between the home-located patient and a hospital-based team *vs *a TM centre coordinating all stakeholders involved^43^
Leadership & governance	Coordination pivot	(g)	Main actor responsible for coordinating care delivery between the multidisciplinary team and the patient^46,48,50^
	Shared decision-making culture	(h)	Patient, informal caregivers and multidisciplinary care team members all take part on the care process^40,49,50^
	*Stages* vs *actors’* coordination	(i)	Program coordination is achieved between places of activity *vs *between participants in the activity^43^
Workforce	Multidisciplinary core workforce	(j)	Informal caregivers, nurses, GPs, and central condition specialists are the main responsible actors^30,36,40,43,46,48–50^
	Supporting workforce	(k)	Other specialty physicians (eg, endocrinologists, psychologists, psychiatrists), dieticians, social workers, pharmacists^43,46,49,50^
Financing	Coverage and/or reimbursement model	(l)	Public-private arrangements along the trajectory of illness^50^
			Consider new reimbursement models (eg, *pay-for-performance*, direct fees to TM centres)^30,43^
	*B2B* vs *B2C*	(m)	Financing/payment flow between two businesses vsdirectly with the end-user^43^
Technologies & medical products	ICTs and TM devices	(n)	Integration of synchronous communication services (eg, VC), two-way interface mobile-based apps/patient portals and TM devices^39,48,49^
			Consider recommendations for comprehensive, unobtrusive (eg, BT-enabled devices), intuitive application and device design^30,39^
	Health data centre	(o)	Interoperable information system that allows information sharing with all the relevant actors^49,50^
Information & research	Health indicators measurement	(p)	For example, BP, heart rate, ECG, weight, glycaemia, dyspnoea, blood oxygenation, sleep patterns, anxiety, physical activity, e-PROs, symptom scores^36,40^
	Clinical dashboards	(q)	Allows a health status comprehensive monitoring in a user-friendly manner^39,50^
			Allows monitoring several patients at the same time^43^
			Access shall be granted to all stakeholders involved, to allow shared decision-making^40^
Monitoring	Outcome measurements	(r)	For example, admissions/readmissions, ED visits, inpatient LoS, all-cause mortality, HRQoL, patient satisfaction, SUS, cost savings, cost/benefit analysis, ROI^36,39^

Abbreviations: RPM, Remote patient monitoring; SELFIE, **S**ustainable int**E**grated chronic care mode**L**s for multi-morbidity: delivery, **F**Inancing, and performanc**E**; TM, telemonitoring; H2H, hospital-to-home; 2HT, high-touch-high-tech; GP, general practitioner; VC, videoconferencing; BT, Bluetooth; BP, blood pressure; ECG, electrocardiogram; e-PROs, electronic patient reported outcomes; ED, emergency department; LoS, length of stay; SUS, system usability score; HRQoL, health-related quality of life; ROI, return on investment.

###  Real-Life Initiatives of Integrated Care Implementation of Remote Patient Monitoring

 Considering the elements of RPM integrated care implementation identified in Table 1, we assessed the extent to which the 19 real-life initiative studies followed the good practices and/or recommendations defining each element. Table 2 presents the conducted assessment, where studies are classified as fully, partially, or not complying with Table 1’s good practices. To illustrate, a study is “fully compliant” regarding the *shared decision-making culture* element if patients, caregivers, and all care team members take part on decision processes; “partially compliant” if patients and/or caregivers are not part of the decision-making process but exists a shared decision-making culture within the multidisciplinary care team; “not compliant” if good practice is not followed. For elements defined by multiple recommendations, failing to address any of them constitutes partial compliance, and failing to address all recommendations is a case of non-compliance.

**Table 2:** Analysis of 19 Real-Life Initiative Studies Regarding the Extent to Which Each Study Entails the Elements Identified for RPM Integrated Care Implementation

**First Author, Year, Country**	**Target Population (and Study Design)**	**Individual & Environment**		**Service Delivery**			**Leadership & Governance**				**Workforce**		**Financing**		**Technologies & Medical Products**		**Information & Research**		**Monitoring**	**Applied Technologies**
		**(a)**	**(b)**	**(c)**	**(d)**	**(e)**	**(f)**	**(g)**	**(h)**	**(i)**	**(j)**	**(k)**	**(l)**	**(m)**	**(n)**	**(o)**	**(p)**	**(q)**	**(r)**	
Agarwal et al, 2021, Canada^47^	COVID-19 (Descriptive study)	∘	∗	∗	∘	●	H2H	●	∗	S	∗	●	∘	B2B	●	∘	●	●	●	Telephone and/or VC + Messaging service + Patient portal (to collect e-PROs) + TM devices (ie, pulse oximeter, thermometer) + EMR + e-learning + Dashboard
Black et al, 2014, USA^29^	HF (RCT)	∘	●	●	∘	∘	H2H	∗	∘	S	●	∗	∗	B2B	∗	∗	●	∘	●	Telephone + BT-enabled TM devices (ie, scale, BP monitor) + EMR
Brooks et al, 2013, USA^35^	PTSD (Program assessment)	●	●	●	∘	∗	2HT	●	●	A	●	●	∘	-	∗	∘	∗	∘	∗	VC + Patient portal (to collect e-PROs) + EMR + e-learning
Casale et al, 2021, USA^11^	COVID-19 (Program assessment)	∘	∗	●	∘	∗	H2H	●	∗	S	●	●	∗	B2B	∗	∗	●	∘	∘	Telephone + TM devices (ie, pulse oximeter, BP monitor, thermometer) + EMR
Cheville et al, 2018, USA^37^	Cancer and hematologic conditions (RCT)	∘	∗	∗	∗	∘	2HT	●	∗	A	●	●	∘	-	●	∗	●	∗	●	Telephone and/or VC + Patient portal (to collect e-PROs) + Telerehabilitation + Pedometer + EMR + e-learning
Dontje et al, 2021, Netherlands^44^	ALS (Participatory action study)	●	●	●	∘	∘	H2H	∗	∘	S	●	∘	∘	B2B	∗	∗	∗	∗	●	Messaging service + Patient portal (ie, mobile app, to collect e-PROs) + Weighting scale + EMR
Fairbrother et al, 2013, Scotland^54^	HF (Qualitative study)	∘	∘	∗	∘	∘	H2H	∘	∘	S	∗	∘	∘	B2B	∗	∘	●	∘	∘	Telephone + Patient portal (to collect e-PROs) + BT-enabled TM devices (ie, pulse oximeter, BP monitor, scale) + e-learning
Foster et al, 2021, USA^34^	Infants with SVP (Program assessment)	∘	∗	●	∗	∗	H2H	●	∗	S	●	∗	∗	B2B	●	∘	●	●	●	VC + Patient portal (ie, mobile app, to collect e-PROs and for video/photo sharing) + TM devices (ie, scale and pulse oximeter) + EMR + e-learning + Dashboard
Herkert et al, 2020, Netherlands^45^	HF + COPD (Quasi-experimental study)	●	●	●	●	●	H2H	●	∗	S	●	∘	∘	B2B	●	∘	●	●	●	Telephone and VC + Messaging service + Patient portal (to collect e-PROs) + BT-enabled TM devices (ie, BP monitor, pulse oximeter, scale, and thermometer) + EMR + Dashboard
Krenitsky et al, 2020, USA^14^	COVID-19 + prenatal care (Program assessment)	∘	∘	∗	●	∘	H2H	∘	∘	S	●	∘	∘	B2B	●	∗	●	∘	∗	VC + Patient portal (to collect e-PROs) + BT-enabled TM devices (ie, BP monitor, pulse oximeter, and thermometer) + EMR
Pelletier et al, 2011, USA^33^	Diabetes (Program assessment)	∘	∗	∗	∘	∘	H2H	∗	∘	S	●	∗	●	B2B	∗	∘	∗	●	∗	Messaging service + Patient portal (for self-care enhancement) + TM devices (ie, glucometer and BP monitor) + Dashboard
Realdon et al, 2018, Italy^41^	Dementia (Program assessment)	∘	∗	●	∗	∗	H2H	∘	∘	S	●	∘	∗	B2B	●	●	●	●	∘	Messaging service + Patient portal (to collect e-PROs) + BT-enabled TM devices (ie, BP monitor, pulse oximeter, thermometer, scale, ECG, glucometer) + Smartwatch + Telerehabilitation + CRM + EMR–e-learning + Dashboard
Ricci and Morichelli, 2013, Italy^42^	Patients with CIED (Program assessment)	∘	∗	●	∘	∘	2HT	●	∗	A	●	∘	∘	B2C	∗	∘	●	∘	●	Telephone and e-mail + Patient portal (for data entry) + (Wireless) CIED
Schenkel et al, 2020, USA^38^	Lung transplant patients (Program assessment)	∘	∗	●	●	∗	2HT	●	∗	A	●	∘	∗	B2C	●	∘	●	∗	●	VC + Patient portal (to collect e-PROs) + BT-enabled TM devices (ie, BP monitor, pulse oximeter, scale, glucometer, spirometer) + EMR + e-learning
Schmidt et al, 2018, Germany^52^	Chronic cardio-vascular diseases (RCT)	∘	∗	●	∘	∗	2HT	●	∗	A	●	∘	∘	B2C	●	∗	∗	●	●	Telephone + Patient portal (to collect e-PROs) + TM devices + EMR + e-learning + Dashboard
Sheeran et al, 2011, USA^32^	Depression (Feasibility study)	∘	∗	●	∘	∘	H2H	●	∗	S	●	∘	∘	-	∗	∘	∗	∘	●	Telephone + Patient portal (to collect e-PROs) + BT-enabled TM devices (ie, BP monitor, pulse oximeter, scale, ECG, glucometer) + e-learning
Singh et al, 2011, USA^31^	Post-acute care (Longitudinal case study)	∗	●	●	∗	∗	2HT	●	●	A	●	∘	●	B2C	∗	●	●	●	●	Telephone + TM devices (ie, BP monitor, pulse oximeter, thermometer, scale, ECG, spirometer) + EMR + Dashboard
Smaradottir et al, 2017, Norway^53^	COPD (Program assessment)	∘	●	●	∘	∘	2HT	∗	∘	A	∗	∘	●	B2C	∗	∗	∗	●	∗	Patient portal (to collect e-PROs) + Pulse oximeter + Dashboard
Vuorinen et al, 2014, Finland^51^	HF (RCT)	∘	∗	●	∘	∗	H2H	∗	∗	S	●	∘	∘	B2B	●	∗	●	∘	●	Telephone + Patient portal (ie, mobile app, to collect e-PROs) + TM devices (ie, BP monitor, scale) + EMR

Abbreviations: RPM, Remote patient monitoring; H2H, hospital-to-home; TM, telemonitoring; VC, videoconferencing; EMR, electronic medical record; B2B, business-to-business; BT, Bluetooth; BP, blood pressure; HF, heart failure; RCT, randomized controlled trial; PTSD, post-traumatic stress disorder; 2HT, high-touch-high-tech; e-PROs, electronic patient reported outcomes; SVP, single ventricle physiology; ALS, amyotrophic lateral sclerosis; CIED, cardiac implantable electronic devices; COPD, chronic obstructive pulmonary disease; B2C, business-to-consumer; CRM, customer relationship management tool. *Notes:* ● Fully complying with Table 1; ∗ Partially complying with Table 1; ∘ Not complying with Table 1; A: actors’ coordination; S: stages’ coordination; (*a*) collaborative design process; (*b*) patient-centred implementation; (*c*) patient education and self-monitoring promotion; (*d*) multi-morbidity care; (*e*) dynamic trajectory of illness; (*f*) H2H vs 2HT; (*g*) coordination pivot; (*h*) shared decision-making culture; (*i*) stages vs actors’ coordination; (*j*) multidisciplinary core workforce; (*k*) supporting workforce; (*l*) coverage and/or reimbursement model; (*m*) B2B vs B2C; (*n*) ICTs and TM devices; (*o*) health data centre; (*p*) health indicators measurement; (*q*) clinical dashboards; (*r*) outcome measurements.

 Most studies referred to, at least, 10 elements of integrated care implementation of RPM (n = 15), in full or partial compliance with Table 1. 12 studies reported a H2H service delivery model, associated to a *stages*’ coordination context. 11 studies complied with a B2B business model, 5 with a B2C and in 3 it was not sufficiently clear.

 Four elements were present in all studies: *patient education and self-monitoring promotion* (13 for full, 6 for partial compliance), *multidisciplinary core workforce* (15 full, 4 partial), *ICTs and TM devices* (9 full, 10 partial), and *health indicators measurement* (13 full, 6 partial). Combining these four elements suggests the elementary design of an RPM intervention, based on a clinical team responsible for monitoring patients’ health status and educating them on correctly operating TM devices to carry out biomedical data collection. *Patient-centred implementation* (n = 17), *coordination pivot* (n = 16), and *outcome measurements* (n = 16) are also present in most studies. Adding these three elements to the previous four allows us to outline a design that meets Leijten et al^26^ definition of integrated care (ie, structured efforts to provide coordinated, proactive, person-centred, multidisciplinary and collaborative care, with transversal performance monitoring). Elements such as *collaborative design process* (n = 4), *multi-morbidity care* (n = 7), *supporting workforce* (n = 7), and *coverage and/or reimbursement model* (n = 8) were present in less than half of the studies, and, when present, mostly were only partially complying with Table 1. Although constituting value-added contributions to the design of interventions, the presence of these elements was not fundamental to consider these initiatives as integrated care implementations of RPM.

**Figure 2:**
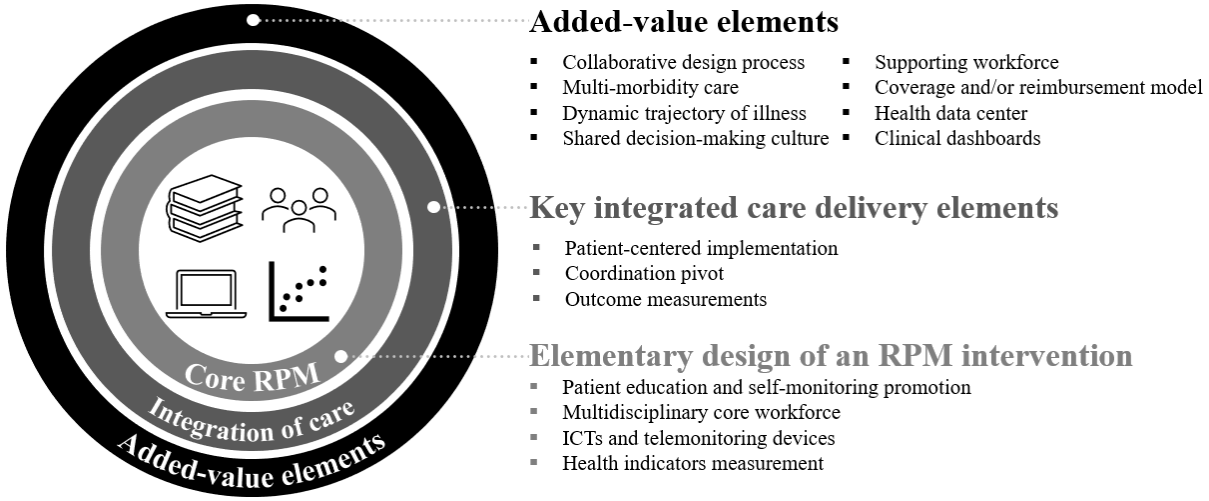
Three-Tier Model for Implementing an RPM-Based Integrated Care Initiative, According to the Structural Elements Identified in Conceptual Models’ Literature and Based on the Results of Real-Life Initiatives’ Analysis. Abbreviations: RPM, Remote patient monitoring; ICTs, information and communication technologies.

Figure 2 summarizes the results of the scoping review, organizing the structural elements identified in Table 1 according to a three-tier model for implementing an RPM-based integrated care initiative, informed by the results of the assessment of real-life initiatives (presented in Table 2) – (1) *elementary design of an RPM intervention*, (2) *key integrated care delivery elements*, and (3) *added-value elements*.

## Discussion

###  General Considerations

 Throughout this scoping review, scientific literature published on conceptual models, and real-life initiatives of RPM integrated care implementation was examined, allowing a thorough identification and assessment of key concepts addressed in this field over the past decade. Included studies comprise a broad diversity of diseases, conditions or target populations (16 different, mostly cardiovascular and/or chronic conditions), as well as a wide geographic dispersion (studies cover 10 different European and North American nations), representing a relevant sample regarding the clinical, cultural, and economic contexts of intervention.

 Applying the SELFIE framework^26^ as a schema for appraisal of included studies ensured the assessment of the main structural components of integrated care delivery. While this framework was developed specifically for the context of multi-morbidity and most RPM initiatives focus on a target condition, we consider that RPM requires an integrated care delivery that should encompass altogether the global health context of the patient ie, associated comorbidities, nutrition and mental care, and social environment, making this framework more suitable for our study’s context (in comparison to other general integrated care models).

 Within SELFIE’s integrated care components, 18 elements of RPM integrated care delivery emerged among analysed conceptual model studies. As a result of a greater thematic focus on aspects related to patient-centred design and coordination between involved actors, the elements associated with *Individual *&* Environment*, *Service delivery* and *Leadership *&* Governance* components are found in equal numbers in relation to the remaining ones. In contrast, elements associated with *Financing* and *Monitoring* are not very representative when analysing the literature on conceptual models. Regarding the first, issues related to financing and reimbursement models in RPM were rarely addressed topics in reviewed studies, so it could be beneficial to explore other sources eg, economic-financial and/or “grey” literature. Guidelines for coverage and reimbursement of healthcare services based on remote care delivery are still not well defined, although there has been progress during the pandemic, namely in coverage for telemedicine services by the Medicare and Medicaid systems in the USA^8,56-58^ and Australia.^59^ As for the *Monitoring* component, there was a greater preponderance of this theme in real-life initiatives’ analysis, with the outcomes to be measured being context-dependent and aligned with interventions’ objectives.

###  Key Messages on RPM Integrated Care Delivery

 This study generates insights for RPM implementation that go far beyond technology. One can interpret that RPM implementation must be a co-creation process between all involved actors, focused on the patient’s needs and encouraging comprehensive and coordinated care delivery. It must address the patient as a complex biological and social system, involving the persons themselves, their caregivers, community, and environment, and not just health professionals and hospital services. Within this view, technology becomes a facilitator of collecting and sharing information (through ICTs and data centres), enabling coordination (through ICTs and dashboards), and permitting evidence-based actions and continuous improvement (through dashboards and outcome measurement).

 The assessment of real-life initiatives showed that most included studies already present a significant degree of care integration, with 78.9% referring to at least 10 of the 18 identified structural elements. Despite being an interesting result, it is not unexpected, as failing to present an integrated care RPM program was an exclusion criterion from the screening process.

 Although elements such as *patient-centred implementation* and *ICTs and TM devices* are present in most studies, they often partially comply with the good practices and/or recommendations from Table 1. Only 8 studies were able to develop truly patient-centred interventions ie, that consider the patient’s needs, preferences, and environment in their fullness. Culture and economic context adaptation of the intervention, and clear definition of informal caregivers’ roles are still issues that need further development. Lack of caregivers’ roles definition also impacts on *shared decision-making culture*, as studies fail to include patient and caregiver in the decision-making process. Likewise, although *ICTs and TM devices* is one of four unanimous elements across studies, 10 only partially comply with Table 1 definition. Programs may not incorporate TM devices (ie, monitoring of e-PROs and symptoms, but not vital signs), fail to provide communication channels between patient/caregiver and provider (eg, patient portals, VC) or may not integrate collected TM data into the EMR. Nevertheless, patient portals (84.2% of real-life initiative studies), EMR (73.7%), telephone, BP monitors, pulse oximeters (57.9% each) and weighting scales (52.6%) are technologies mentioned in most studies, reinforcing the role of technology in allowing (*a*) synchronous and asynchronous communication, (*b*) monitoring the patient’s health status, and (*c*) data recording and sharing with all involved actors. Other elements, such as *dynamic trajectory of illness* and *health data centre*, also exhibit high levels of partial compliance, thus new projects should consider including them.

 Besides *coverage and/or reimbursement model* (already discussed above), *collaborative design process*, *multi-morbidity care*, and *supporting workforce* are present in less than half of the studies. Stakeholder involvement in the design process appears to correlate with developing patient-centred solutions, as the three studies that fully comply with *collaborative design process* are also fully compliant with *patient-centred implementation*. Furthermore, lack of *multi-morbidity care* and *supporting workforce* elements across studies suggest a devaluation of their importance in improving care, as it may add a complexity layer that does not necessarily translate into added value.

 Last, but not least, even though an assessment of implemented initiatives was carried out, it was not intended to develop correlations between intervention complexity and its effectiveness in care provision. The latter largely depends on the target condition of intervention, the patient groups included, and the objectives outlined for it. However, for comparable implementation scenarios, it is our expectation that an intervention comprising a greater number of structural elements will lead to better clinical, social, and economic outcomes. Further studies should be conducted to respond to this hypothesis.

###  Implications for Practice and Future Research

 The structural elements identified and the good practices and/or recommendations that define them should contribute to the development and organization of care integrated models in RPM. By highlighting the contribution of these elements to a more patient-focused, more efficiently coordinated, and more attentive implementation, this review deepens our understanding of the added value of remote care initiatives in delivering more needs-driven healthcare, optimizing resource allocation, and improving the overall experience and quality of life of the patient.

 The development of a dissemination strategy for RPM should be an active policy-making process, involving different health sectors (eg, primary, secondary, tertiary), whether public, private, or social, and may also include religious institutions, employers, housing, local communities, and education. Furthermore, for technology providers and implementation partners, the results of this scoping review can inform about the perspectives of patients (and caregivers) and health professionals regarding which features should be considered in developing more end-user-centric ICT technologies.

 Moreover, this study has shown that literature combining RPM and care integration is still scarce and novel studies to inform its implementation are in need. An increased focus by researchers on legislation and policy for remote care provision can inform the implementation of newly designed RPM programs and help to better predict their applicability, feasibility, and success in specific geopolitical contexts.

 Additionally, the RPM care integration elements identified throughout this study can be leveraged as a starting point for developing a systematic review of RPM initiatives or comparative study between RPM interventions, aiming at developing comprehensive assessments of the relationship between completeness in intervention design and its effectiveness, measured by clinical criteria and non-clinical outcomes.

###  Limitations

 While efforts have been made to assure methodological thoroughness, this scoping review is not without limitations. Despite conducting a comprehensive search strategy, which considered terminological variations and alternative spellings of search keywords, the terms “telehealth” or “telemedicine” were not included, which may have led to the non-identification of relevant studies. The decision not to include these terms was the result of the trade-off between how many relevant *versus* non-relevant studies would have been identified, concluding that it was preferable not to include, given the time and resource limitations in the development of this work.

 From our perspective, and as already discussed, using the SELFIE framework positively contributed to the appraisal of the integrated care nature of RPM studies. However, and as identified by the authors of the framework,^22^ topics related to the use of eHealth, although believed to have great potential for improving integrated care for multi-morbid patients, are scarce in multi-morbidity literature. Thus, the use of the SELFIE framework for the RPM context may present limitations, as most literature that informs the framework’s development does not consider a digital-first care delivery setting.

 As already mentioned, the lack of elements concerning *Financing* could be resolved by including economic-financial or “grey” literature in the search protocol. Additionally, although included studies present an enriching diversity regarding the diseases, conditions or target populations addressed, this also poses a challenge in the aggregation and appraisal of results, since the elements identified as structural in RPM care integration can acquire greater importance in certain clinical contexts.

## Conclusion

 The overall aim of this study was to provide a comprehensive analysis of what structural elements should be considered in the implementation of RPM solutions that are aligned with a logic of care integration. Based on literature referring to conceptual models and real-life initiatives of RPM, 18 structural elements were identified, described according to good practices and recommendations that guide their implementation, and organized into a three-tier model, based on the practical contribution of each element within the scope of implementation.

 We hope that our work will inform future decisions about the implementation of RPM services in healthcare systems and that it can help decision-makers ensuring that key elements for RPM proper implementation are ensured, thereby contributing to improved cost-effective adoption, and better outcomes for patients, providers, and society at large.

 As a final remark to all stakeholders responsible for driving the dissemination of remote healthcare, the COVID-19 pandemic has contributed to unlocking some barriers to RPM implementation and one should consider multiple elements when developing robust initiatives, that are more planned and more able to rapidly overcome pilot phases, and which can serve efficiently and with quality increasing numbers of patients.

## Acknowledgements

 RM would like to acknowledge Siemens Healthineers and Centro de Estudos de Gestão of Instituto Superior Técnico (a research centre funded by FCT project UIDB/00097/2020), host institutions for the doctoral research this study is part of.

## Ethical issues

 Not applicable.

## Competing interests

 This work is an integral part of RM’s doctoral research, carried out in collaboration between Instituto Superior Técnico (University of Lisbon) and Siemens Healthineers. Co-author FMB (PhD Epidemiology and Public Health) is an employee from Siemens Healthineers, and we naturally state that the developed work had a scientific nature and complied with ethical norms.

## Authors’ contributions

 RM: Conceptualization, methodology, formal analysis, investigation, data curation, writing-original draft, and visualization. MDO: Methodology, validation, formal analysis, writing-review & editing, and supervision. PN: Methodology, resources, and writing-review & editing. FMB: Writing-review & editing and supervision. IA: Writing-review & editing and supervision.

## Funding

 RM’s research was supported by the doctoral scholarship 2020.05845.BD granted by the Portuguese Foundation for Science and Technology (FCT) and in connection with the MEDI-VALUE research project (FCT grant no. PTDC/EGE-OGE/29699/2017). MDO’s work was developed within the scope of the MEDI-VALUE project.

## Supplementary files


Supplementary file 1 contains Table S1.
