# Supplementary material for: Towards A Framework for Implementing Remote Patient Monitoring From an Integrated Care Perspective: A Scoping Review
Source: Int J Health Policy Manag. 2023 Mar 1;12:7299. doi: 10.34172/ijhpm.2023.7299 (PMC10461888; doi:10.34172/ijhpm.2023.7299)
Supplement: Supplementary file 1 — contains Table S1. [file ijhpm-12-7299-s001.pdf]

Article title: Towards A Framework for Implementing Remote Patient Monitoring From an Integrated Care Perspective: A Scoping Review

Journal name: International Journal of Health Policy and Management (IJHPM)

Authors' information: Rafael Miranda[1,2]*, Mónica Duarte Oliveira[1,3], Paulo Nicola[4], Filipa Matos Baptista[2], Isabel Albuquerque[5]

[1]Centro de Estudos de Gestão do Instituto Superior Técnico (CEG-IST), Instituto Superior Técnico, Universidade de Lisboa, Lisboa, Portugal.

[2]Enterprise Services, Siemens Healthineers Portugal, Lisboa, Portugal.

[3]iBB - Institute for Bioengineering and Biosciences and i4HB - Associate Laboratory Institute for Health and Bioeconomy, Instituto Superior Técnico, Universidade de Lisboa, Lisboa, Portugal.

[4]Instituto de Medicina Preventiva e Saúde Pública, Faculdade de Medicina, Universidade de Lisboa, Lisboa, Portugal.

[5]Comprehensive Health Research Center (CHRC), Universidade NOVA de Lisboa, Lisboa, Portugal.

(*Corresponding author: rafaelpiresmiranda@tecnico.ulisboa.pt)

Supplementary file 1

Table S1. Rules for scoping review search protocol: [("A" or "B") and ("C" or "D" or "E") and ("F" or "G" or "H" or "I")].

A: concept* OR theor* OR framework* OR model* OR plan*<br>B: program* OR action* OR project* OR approach* OR initiative* OR implement*<br>C: "remote patient monitoring" OR "remote monitoring" OR "remote care" OR "home monitoring"<br>D: telemonitor* OR telesurveillance OR telemetric<br>E: tele-monitor* OR tele-surveillance OR tele-metric<br>F: "care integration" OR "integrated care" OR "integrated care delivery" OR "care delivery" OR "health care delivery" OR "integrated health care delivery" OR "integration of care" OR "delivery of health care integrated" OR "integrated medicine" OR "clinical integration"	G: "care continuity" OR "care continuation" OR "continuity of care" OR "continuity of patient care"<br>H: "patient-centred" OR "patient-centered" OR "patient-centred care" OR "patient-centered care"<br>I: "managed care" OR "patient care management" OR "management model" OR "comprehensive care" OR "comprehensive health care" OR "care coordination" OR "collaborative care" OR "shared care" OR "accountable care" OR "multidisciplinary care" OR "interdisciplinary care" OR "inter-disciplinary care" OR "transmural care" OR "holistic care"
